# Main-Group Metal Complexes of Benzene: Predicted Features of Stabilization and Isomerization

**DOI:** 10.3390/molecules28165985

**Published:** 2023-08-10

**Authors:** Fedor Y. Naumkin

**Affiliations:** Faculty of Science, Ontario Tech University (UOIT), Oshawa, ON M4W 3C8, Canada; fedor.naumkin@uoit.ca

**Keywords:** intermolecular complexes, metal–organic compounds, aromaticity, ab initio calculations

## Abstract

Complexes of metal atoms with organic molecules represent a broad variety of systems with many important applications, e.g., in metal–organic interfaces and organometallic chemistry. One class involves aromatic species like benzene (Bz). Here, such complexes with second-group metals are investigated systematically in terms of structure and shape, stability and isomerization, charge distribution and aromaticity, and polarity and IR spectra. Three groups of isomers are identified, varying from metastable to stable ones, in effect featuring “physisorption” or “chemisorption”. In particular, the high polarity of binary complexes and nonadditive stabilization of ternary systems for some isomers are found. Also, the Bz component’s shape alteration for different isomers and system sizes and related aromaticity variations are predicted to be considerable. Property evolution for the series of metal components is analyzed.

## 1. Introduction

Nanointerfaces between metals and organic molecules represent a variety of systems involved in multiple applications based on catalysis, light–matter interactions, property modifications, etc. In particular, organic ligands can enhance and/or tune (e.g., in terms of selectivity) the catalytic activity of metal centres in inorganic complexes. The optical properties of metal nanospecies can be altered by their adsorption on a molecular fragment of, for instance, a fullerene, graphene, or a carbon nanotube. In turn, the inherent high polarizabilities of metal atoms can improve, for example, the photoabsorption performance of molecular hosts. Interactions between molecules and metal atoms can also change the shape and/or structure of the former and/or link a few of them into a larger entity, and in this way, possibly affect their other properties as well.

Among a huge multitude of such systems, intermolecular complexes of second-group metal atoms (mainly Be and Mg) were previously investigated from the viewpoint of nonlinear optical properties, such as hyperpolarizability [1,2], in relation to astrochemistry and radioastronomy [3,4], metal-containing heterocycles [5], intra- and intermolecular cooperativity of binding [6], and as potential units for a charge-governed structure manipulation [7]. In particular, one such very uncommon compound, diberyllocene (with a Be-Be bond), was produced in experiments very recently [8]. Extended versions of such systems were analyzed in terms of the formation of mixed metal–hydrocarbon clusters [9], as well as the adsorption of metal atoms and clusters on graphene and graphite surface [10]. In particular, the adsorption of alkaline-earth metal atoms on carbon nanostructures was indicated as capable of improving hydrogen storage capacity [11].

Most relevant earlier theoretical studies involved M-benzene and M-pyridine complexes (M = Be to Ca) [11,12]. Interestingly, weakly bound M-Bz species have been found to be more stable than chemically bonded isomers.

In the present work, metal–benzene binary (M-Bz) and ternary (M-Bz-M) complexes are studied for a complete series of M = Be to Ba. Both weakly bound and chemically bonded isomers and the stabilization of M-Bz, upon the addition of the second M, are considered. Relevant alterations of the Bz-ring shape and the related evolution of its aromaticity and the complexes’ IR spectra are addressed. Due to their structure, these systems, besides their own value for, e.g., organometallic and astrochemistry, could also serve as units (or at least models), representing molecular-scale interfaces between such metal atoms and graphite, graphene, carbon nanotubes, etc. In particular, the attachment of two M atoms on both sides could potentially enhance the function of such interfaces by offering two (possibly interacting) active sites.

## 2. Results and Discussion

### 2.1. Structures and Stabilities

**Figure 1:**
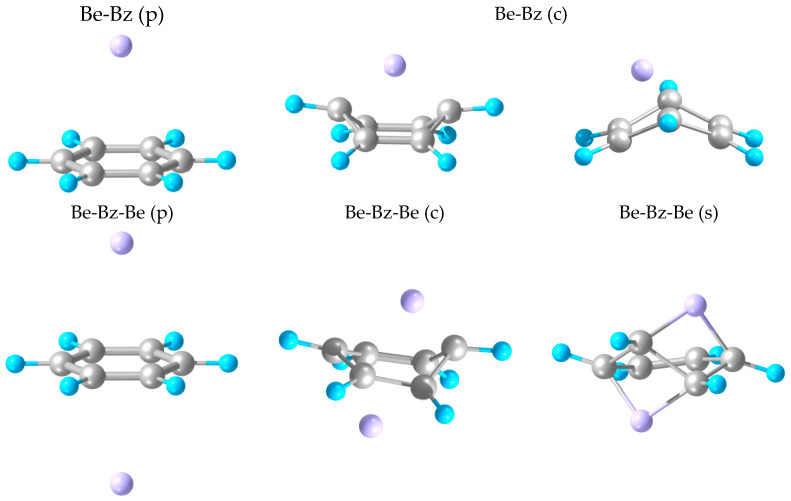

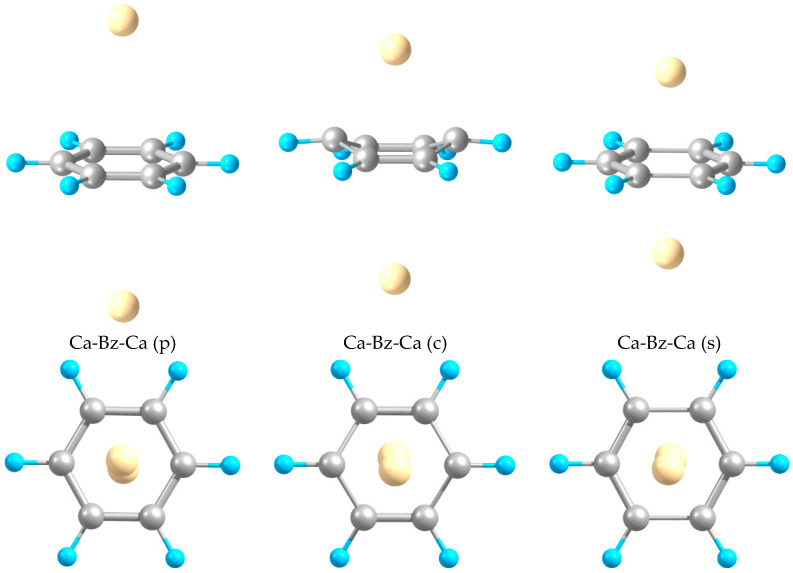
Optimized geometries of Be-Bz “physi-” (p) and “chemisorbed” (c), Be-Bz-Be, and (front and top views) Ca-Bz-Ca complexes—p-, c-, and s-isomers.

Attachment of an M atom (M = Be to Ba) axially to Bz leads to common M-Bz “half-sandwiches” (Figure 1). The complex’s stability generally increases with the metal atom’s size, approximately doubling from Be to Ba (Table 1), while remaining relatively weak (with a dissociation energy, D_e_, under 0.2 eV). This could be viewed as molecular-level physisorption (hence, p-isomer for short, hereafter) and is in accordance with the closed-subshell electronic structure of the attached atoms. The M-Bz distance, however, reaches a maximum value at M = Ca, probably reflecting the interplay between the increasing size of M and its increasing attraction to Bz. The obtained D_e_ values for M = Be to Mg are close (within about 30% for M = Be and 10% for M = Mg, Ca) to those predicted previously [11] at a higher CCSD(T) level of theory.

Another isomer corresponds to M bonding to two opposite C atoms of Bz and to the resulting partial folding of the Bz ring (Figure 1) such that C-C bond lengths slightly vary (Table 1) in accordance with the only two remaining double bonds. Such folding is not found for M = Mg (no such isomer obtained), is the strongest for M = Be (by about 50°), and drops significantly (to about 30°) and increases for heavier M. The M-C distances shrink considerably (decreasingly for heavier M), which, together with the apparent alteration from the sp^2^ to sp^3^ coordination of the two involved C atoms, can be formally associated with molecular chemisorption (hence, c-isomer hereafter). For M = Be and Ba, the M-Bz binding is stronger than for the previous isomer (up to about twice). However, it produces slightly metastable (higher in energy than separated M and Bz) systems for M = Ca and Sr such that strain in the distorted Bz ring exceeds stabilization due to the bond formation for these cases. In particular, this strain can be evaluated as the energy difference between the Bz-component frozen in its geometry in M-Bz and the free Bz molecule, giving 2.9 eV for M = Be and 1 ± 0.1 eV for M = Ca to Ba. Additionally, Be is shifted to one side of the (part-folded) Bz ring by about 0.7 Å in terms of Be-C distances (to the double bonded C atoms), unlike the heavier M atoms remaining in its axis. This leads to the higher coordination of Be to Bz, likely contributing to the stability of Be-Bz, in spite of the largest strain in the Bz component for this case. The higher stability of the p-isomers for M = Mg and Ca confirms the earlier predictions [11]. The same is valid for the c-isomer geometry with an axial location of Be, while, here, it represents a transition state.

The calculated potential energy barrier between the two isomers, from the “physisorbed” side, decreases from about 0.3 eV for Be to about 0.1 eV for Ba. Accordingly, the corresponding reverse barriers stabilizing metastable “chemisorbed” Ca-Bz and Sr-Bz are low at about 0.1 eV. Curiously, for Be-Bz and Ba-Bz the “physisorption” state is, thus, in effect, metastable, with respect to “chemisorption”.

For the p-isomer of M-Bz, the attachment of the second M on the opposite side of Bz leads to a symmetric “physisorbed” M-Bz-M structure (Figure 1), with each M atom slightly (by 0.1–0.2 Å) farther away from Bz than in M-Bz. The latter could perhaps be interpreted in terms of a repulsion of oppositely directed dipoles induced on M by Bz. The M-C distances repeat the variation shown by M-Bz, i.e., increase from M = Be to Ca and then drop for Sr and Ba. Consequently, the same trend is followed by the M-M distance. Accordingly, the M-Bz-M dissociation energy is approximately doubled compared to that for M-Bz, except for M = Ba, in which case it exhibits only a slight increase in D_e_. The latter uncooperative nonadditivity of interactions for M = Ba appears to be consistent with the decreased M-M distance and increased Bz-induced dipoles on M (as compared to lighter M), together leading to their stronger repulsive contributions.

For the c-isomer of M-Bz (absent for M = Mg), a similar addition of another M can result in a corresponding asymmetric M-Bz-M c-isomer, with the original M-Bz unit preserving part-folded Bz and the second M farther away from it (Figure 1). In particular, such a system acquires a somewhat distorted shape (with the Bz ring folding more on one side) for M = Be, unlike for heavier M. Furthermore, such a Be-based ternary complex has a D_e_ that is nearly identical to that of the original Be-Bz unit, as well as with the p-isomer of Be-Bz-Be (Table 1), which is likely related to an increased strain in the additionally distorted Bz ring. In contrast, for M = Ca and Sr, the c-isomer of M-Bz-M is more symmetric and stabilized relative to M-Bz by about 0.2 and 0.3 eV, respectively. This reduces the metastable character of the binary complex for the former or even makes it slightly stable for the latter case. No such isomer is found for M = Ba, however.

Additionally, the above c-isomer of Be-Bz-Be is nearly a transition state with only a tiny barrier separating it from the more stable (by about 1 eV) s-isomer. The latter has the C_6_ ring with two pairs of C atoms bridged by Be atoms (Figure 1) so that just one double C=C bond remains, while the opposite side of the ring stretches and zigzags characteristically of the sp^3^ coordination pattern. We refer the reader to Table S1 in Supplementary Materials for geometrical details.

In contrast, a heavier M-Bz-M (except for M = Mg) exhibits a symmetric s-isomer with a flat Bz ring, which has two opposite C-C bonds stretched by about 0.1 Å. The system is also significantly compressed axially, i.e., with a shorter M-M distance as compared to the ternary c-isomer—by up to 1 Å for M = Ca and Sr. Relative to the p-isomer, the axial compression is more significant and ranges from about 2.7 (i.e., almost by half for M = Be) to about 1.3 Å (for Ba). Compared to “physisorbed” M-Bz-M, such s-isomers show stability (in terms of D_e_), varying from half for M = Ca to almost the same for Sr to about sixfold for Ba and Be, thus demonstrating the uncooperative to strongly cooperative nonadditivity of interactions.

In particular, the c-isomer of Ca-Bz-Ca is separated from its s-isomer by a low potential energy barrier of about 0.1 eV. A similar barrier is obtained for the axial compression of the p- into the s-isomer of Ba-Bz-Ba. The latter barrier is thus about the same as that for the p- to c-isomerization of Ba-Bz.

### 2.2. Charge Distributions and Polarities

The increasing (with M size) attraction between M and Bz in “physisorbed” M-Bz is likely related to the increasing polarization of M (due to its increasing polarizability) by the electric field of the quadrupole moment of Bz. Indeed, the dipole moment of M-Bz is significant and increases from Be to Ba (Table 2), being directed from M to Bz, and is consistent with its induction by the quadrupole field of Bz. This is further supported by the fact that both components of the complex are found to carry essentially no charges, indicating that the dipole is apparently not due to charge transfer.

The formation of M-C bonds in the c-isomer facilitates a considerable charge transfer from M to Bz (Table 2), mainly to the C atoms, increasing with the decreasing ionization energy of M from Ca to Ba. The relatively high charge on Be could be related to its higher coordination (to more C atoms). The dipoles correlate with the charges and are now directed from Bz to M and, hence, opposite to the previous case. In particular, a simple estimate as a product of the charge on M and the axial M-Bz distance gives a large value of about 8 to 16 D for M = Ca to Ba. Nevertheless, the dipole values are smaller than for the p-isomers, especially for Ca and Sr, except for the Be case. Such a non-obvious relation is consistent with the counter-contribution to the total dipole from the part-folded Bz ring (pointing from C to H atoms, hence away from M), as well as from the induced polarization of both components.

For the c-isomer of M-Bz-M (M = Ca and Sr), the attached second M atom remains essentially neutral, while the charge transfer from Bz to the first M atom increases (Table 2). This is more pronounced for M = Ca, even though the charge remains higher in (nearer) Sr. In Be-Bz-Be, both Be atoms are charged, although they are charged somewhat differently and less than in Be-Bz. For the s-isomer, two equivalent M atoms are equally charged, with the charge value being significantly larger for Be and slightly decreasing for M = Ca to Ba. Accordingly, the c-isomer of M-Bz-M is more polar than M-Bz by a factor of about 5 for M = Ca and 4 for Sr. This could be related to the added polarization of the neutral second M by a field-cooperative combination of the Bz quadrupole and M-Bz dipole. In contrast, for M = Be, the dipole is slightly reduced, which could be interpreted in terms of a smaller charge on the first Be atom, plus the counter-contribution from the comparable charge on the second Be (with the corresponding Bz-Be dipole component directed oppositely).

The s-isomer of M-Bz-M is polar only for M = Be due to an asymmetry of its structure. Here, the M atoms are charged about as much as in the c-isomer of Be-Bz.

### 2.3. Aromaticity Evaluation

In the above complexes, the Bz-ring shape evolves from symmetric-flat (in p-isomers of M-Bz and M-Bz-M) to part-folded (in c-isomers of M-Bz, as well as M-Bz-M for M = Ca and Sr) to stretched-flat (in s-isomer of M-Bz-M for M = Ca to Ba) to a low-symmetry distorted shape (for c- and s-isomers of Be-Bz-Be). The corresponding changes in the ring’s aromaticity, an inherent property of free Bz, can be tracked in terms of various dedicated parameters. Here, two of these are chosen—the harmonic oscillator measure of aromaticity (HOMA) and the multi-centre bond order (MCBO), both calculated by means of the Multiwfn software (see Section 3 below).

All “physisorbed” systems exhibit almost unchanged values for both parameters from those of free Bz (Table 2), reflecting the weak influence of M and, hence, a full aromaticity of the ring. For the c-isomer of M-Bz, there are significant deviations, which are the lowest for Sr-Bz (with HOMA being under 50% of the free-Bz value and MCBO reaching 90%) and the largest for Be-Bz (even negative HOMA and MCBO of 70%), consistent with the strongest Bz-folding for the latter case (Table 1). A similar trend can be seen for the corresponding ternary c-isomers, with HOMA even somewhat more negative and a lower MCBO for Be-Bz-Be, while staying nearly the same for the heavier M-Bz-M. The ternary s-isomers show a stronger decrease in aromaticity, with HOMA being negative for all M but Ba and MCBO being the lowest up to negative. Be-Bz-Be again shows the largest drop, consistent with both the Bz flatness and the C-C distance uniformity violated, unlike for the heavier counterparts.

### 2.4. Vibrations and Simulated IR Spectra

**Figure 2:**
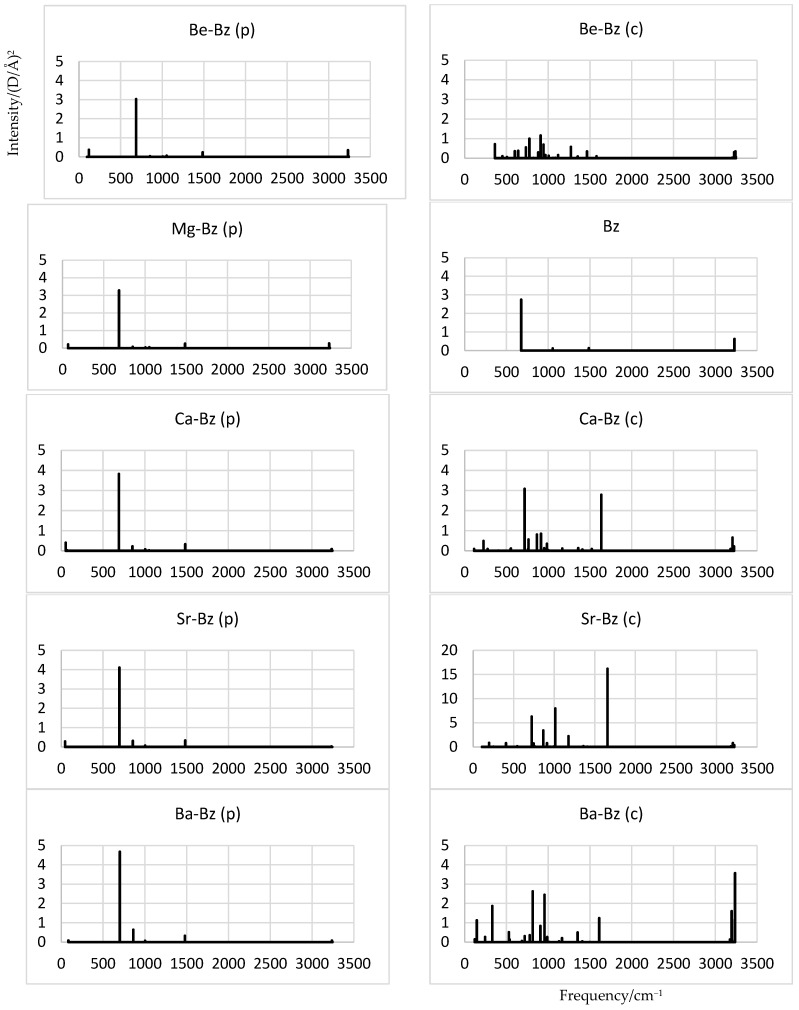
Simulated IR spectra of M-Bz complexes.

The p-isomer of M-Bz exhibits an IR spectrum resembling that of free Bz (Figure 2), as expected in view of a weak interaction between the components. It is thus dominated by the C-H bending vibrations in the axial direction, with the intensity gradually increasing from M = Be to Ba by up to about 50%. The band is located near 700 cm^−1^ and slightly blue shifts (within 30 cm^−1^) with an increasing M size, in accordance with the increasing interaction.

The c-isomer of M-Bz exhibits a few bright bands, and they are considerably less intense for Be-Bz, which is likely due to its lower symmetry and the considerable distortion of Bz. For M = Ca, the above band near 700 cm^−1^ is recovered and accompanied by a near-equally bright band near 1600 cm^−1^, which could be associated with the ring modes of Bz. For M = Sr, the latter band becomes about twice brighter than the former, and the near-700 cm^−1^ band weakens, while another bright band near 1000 cm^−1^ appears, matching the C-H bending vibrations perpendicular to the axis. The latter spectrum is also notably brighter than for other M-Bz, although the origin of this is not clear. And, for M = Ba, a high-frequency band (near 3200 cm^−1^) corresponding to the C-H stretch is uniquely strong among all M-Bz, in effect, at the expense of the (much weakening) near-1600 cm^−1^ band. Still, another low-frequency bright band appears near 300 cm^−1^ as well.

### 2.5. Other Electronic States

In order to selectively evaluate the effect of the electronic perturbation on the system properties, the cationic and triplet-state derivatives of the Ba-Bz sample system are considered as well. In particular, the Bz component becomes more aromatic in either case.

For the excited triplet state of the c-isomer, the dissociation energy slightly reduces (to 0.27 eV), the Ba-C distances slightly stretch (to 2.88–2.95 Å), and the Bz ring unfolds to 171°. All these variations are in accordance with a weaker bonding. As a result, HOMA = 0.839 and MCBO = 0.591, indicating significantly higher aromaticity compared to Ba-Bz (c).

The cationic c-isomer, however, is significantly stabilized to D_e_ = 1.23 eV, even though the Ba-C separation increases to 2.79–2.88 Å, and the folding angle increases to 169°. Here, apparently, the binding in the system is more contributed by the induced polarization of Bz by Ba^+^. For this case, HOMA = 0.833 and MCBO = 0.593 are almost the same as for the triplet-state case, in accordance with the very close folding-angle value.

## 3. Computational Methods and Tools

As an appropriate compromise between the computational efficiency and the need to properly treat relevant non-covalent interactions, the MP2 (second-order perturbation theory) level of theory is employed by means of the NWChem ab initio package [13]. Extensive augmented correlation-consistent (aug-cc-pVTZ) basis sets for lighter atoms (C, H, Be, and Mg) are combined with Stuttgart’s relativistic small-core effective core RSC potentials for heavier metals (Ca, Sr, and Ba) [14].

In view of the anticipated charge-transfer interactions involved, as a test of the chosen approach, the ionization energies of the metal atoms are predicted as 8.83, 7.31, 5.89, 5.47, and 4.92 eV, respectively, for Be, Mg, Ca, Sr, and Ba and compared to the corresponding experimental values of 9.32, 7.65, 6.11, 5.69, and 5.28 eV [15]. The deviations thus do not exceed 7%. Another available test involves the equilibrium distance of CaC, with an experimental value of 2.302 Å [16] favourably matched by the predicted 2.35 Å (deviating within 2%).

All systems are fully optimized with no symmetry constraints, and the energy minima are verified in terms of (all-real) vibrational frequencies. The corresponding IR spectra are produced as well using NWChem. Dissociation energies are calculated in the usual manner (total energy of fragments minus that for the system) and corrected for BSSE (basis-set superposition error) in the standard counterpoise way [17]. Each energy barrier is scanned along a suitable coordinate connecting initial and final structures, with the rest of the geometry reoptimized at each point. Atomic charges are obtained from the natural population analysis (NPA) [18] by means of the JANPA software (version 2.01) [19]. Relevant aromaticity parameters are calculated the HOMA (harmonic oscillator measure of aromaticity [20]) and MCBO (multi-centre bond order [21]), as implemented in the Multiwfn software (version 3.8 dev) [22].

## 4. Conclusions

A series of binary (M-Bz) and ternary (M-Bz-M) complexes of benzene with the second-group metals (M = Be to Ba) are consistently investigated at the MP2 level of theory. The studied species include “physisorbed” (p-isomers), involving relatively weak electrostatic interaction of intact Bz; “chemisorbed” (c-isomers) with bent Bz bonded to M (one of two in M-Bz-M); and the s-isomers of M-Bz-M with the Bz ring remaining flat but stretched along one long diagonal. Some exceptions include Be-Bz-Be with its s-isomer actually having a non-flat Bz ring, Ba-Bz-Ba with a c-isomer absent, and Mg-Bz and Mg-Bz-Mg with only p-isomers found.

The asymmetric c-isomers of both M-Bz and M-Bz-M show stability that is comparable to that of the p-isomers or metastability (with energy above that for separated M and Bz). This could apparently be related to the strain in part-folded Bz. Accordingly, the symmetric s-isomers of M-Bz-M are weakly to significantly bound (by up to 1–1.5 eV). For the latter case, the ternary system exhibits a cooperative nonadditivity of interactions.

The predicted considerable polarity of the M-Bz’s p-isomer is consistent with a Bz-quadrupole-induced dipole on M. For the corresponding c-isomer, the Bz-to-M charge transfer counters the polarization of the components (for Bz—also bonding-induced), reducing the polarity—except for Be-Bz, with its increased coordination of Be in a skewed geometry. The addition of another M fully depolarizes the symmetric p- and s-isomers of M-Bz-M while it strongly increases the polarity of their c-isomers (for M = Ca and Sr). The p- and c-isomers of M-Bz have oppositely oriented dipoles (up to about 5 D for M = Ba), while being separated by relatively low-potential energy barriers (especially for M = Ba). The latter feature is also found for the c- to s-isomerization of Ca-Bz-Ca (with a dipole alteration of about 6 D). This might facilitate some pressure-controlled polarity-switch functions of such molecular systems, like turn-around or turn-off, respectively.

The calculated IR spectra of M-Bz reflect the interactions in the systems, varying weakly for the p-isomers while showing peculiar features for the c-isomers. In particular, such complexations can suppress the C-H vibrations for M = Be, while amplifying the ring-mode bands for M = Ca and Sr or the C-H bands for M = Ba. It is hoped that these features can help the experimental identification of the systems under study. Another way of detecting the highly polar species (especially Ba-Bz(c), M-Bz (p) with M = Ca to Ba) could involve microwave spectroscopy.

The aromaticity of the Bz component appears to be another sensitive detector of its shape variation in (hence the formation of) the complexes. Specifically, the relevant parameters (HOMA and MCBO) are predicted to reduce more strongly due to the ring elongation than its bending. Accordingly, this is more pronounced (up to negative values of the parameters) in the ternary systems, especially in their s-isomers. However, aromaticity can increase upon ionization or excitation to the triplet state, as illustrated for Ba-Bz.

In general, the studied complexes could also serve as structural parts in a huge variety of analogous systems based on molecules with benzene or, e.g., phenol components (or their derivatives). In particular, such systems may be of interest spectroscopically via broadening the ranges of interaction with light covered by the corresponding free molecules, as well as making their spectra more intense. Ultimately, this might potentially be used for more efficient solar-energy utilization, at least at the molecular level, as well as for molecular electronics and machinery.

## Figures and Tables

**Table 1:** Equilibrium parameters (dissociation energies, equilibrium distances, and folding angle) of studied metal–benzene complexes. Here, (p) denotes p-isomer, (c) c-isomer, and (s) s-isomer.

System	D_e_^†^/eV	R_e_ (M-C)/Å	R_e_ (C-C)	φ (C_4_) /deg	R_e_ (M-M)
Be-Bz (p)	0.11	3.13	1.39	180	
Be-Bz (c)	0.20	1.76, 1.81, 2.50	1.35–1.48	130	
Mg-Bz (p)	0.11	3.40–3.43	1.39	180	
Ca-Bz (p)	0.13	3.50–3.51	1.39	180	
Ca-Bz (c)	−0.23	2.48, 2.73	1.37, 1.44	151	
Sr-Bz (p)	0.12	3.41–3.47	1.39	180	
Sr-Bz (c)	−0.22	2.62, 2.86	1.37, 1.44	153	
Ba-Bz (p)	0.20	3.32	1.39	180	
Ba-Bz (c)	0.33	2.64, 2.86	1.37, 1.45	154	
Be-Bz-Be (p)	0.21	3.19–3.20	1.39	180	5.75
Be-Bz-Be (c)	0.20	1.72–2.83	1.37–1.52	135–140	3.29
Be-Bz-Be (s)	1.23	1.64–2.66	1.37–1.65	153	3.01
Mg-Bz-Mg (p)	0.21	3.46–3.48	1.39	180	6.36
Ca-Bz-Ca (p)	0.25	3.66–3.69	1.39	180	6.80
Ca-Bz-Ca (c)	−0.05	2.53/3.55, 2.79/3.25 ^⁑^	1.37, 1.44	150	5.36
Ca-Bz-Ca (s)	0.12	2.61, 2.62	1.40, 1.50	180	4.38
Sr-Bz-Sr (p)	0.25	3.64–3.68	1.39	180	6.78
Sr-Bz-Sr (c)	0.05	2.69/3.52, 2.93/3.24 ^⁑^	1.37, 1.44	153	5.51
Sr-Bz-Sr (s)	0.28	2.75, 2.77	1.40, 1.50	180	4.73
Ba-Bz-Ba (p)	0.25	3.47	1.39	180	6.36
Ba-Bz-Ba (s)	1.40	2.89, 2.91	1.40, 1.50	180	5.05

^†^ D_e_ (M-Bz-M) = 2 E(M) + E(Bz)—E(M-Bz-M), E being total energy. ^⁑^ Pairs indicate distances to the same C atoms from two M atoms.

**Table 2:** Calculated charges (Q) and dipole moments (µ) of studied metal–benzene complexes and aromaticity parameters (HOMA and MCBO) of their Bz components *. Here, (p) denotes p-isomer, © c-isomer, and (s) s-isomer.

System	Q(M)/e	µ/D	HOMA	MCBO
Be-Bz (p)	−0.006	1.70	1.000	0.659
Be-Bz (c)	1.447	2.13	−0.471	0.482
Mg-Bz (p)	0.0	1.95	1.000	0.659
Ca-Bz (p)	0.009	3.32	0.901	0.644
Ca-Bz (c)	0.776	1.28	0.138	0.581
Sr-Bz (p)	0.018	4.19	0.999	0.649
Sr-Bz (c)	0.925	1.67	0.461	0.586
Ba-Bz (p)	0.030	5.16	0.998	0.659
Ba-Bz (c)	1.487	4.98	0.310	0.557
Be-Bz-Be (p)	0.005		0.999	0.656
Be-Bz-Be (c)	1.000, 0.850	1.83	−0.572	0.425
Be-Bz-Be (s)	1.433	0.74	−4.464	−0.400
Mg-Bz-Mg (p)	0.0		0.999	0.661
Ca-Bz-Ca (p)	0.005		0.999	0.635
Ca-Bz-Ca (c)	0.951, 0.027	5.90	0.543	0.582
Ca-Bz-Ca (s)	0.951		−0.178	0.347
Sr-Bz-Sr (p)	0.008		0.998	0.635
Sr-Bz-Sr (c)	0.994, 0.032	6.82	0.589	0.596
Sr-Bz-Sr (s)	0.942		−0.058	−0.349
Ba-Bz-Ba (p)	0.019		0.995	0.642
Ba-Bz-Ba (s)	0.915		0.136	0.259

* For free C_6_H_6_: HOMA = 1.000; MCBO = 0.651.

## Data Availability

The data will be made available upon request.

## Supplementary Materials

The following supporting information can be downloaded at https://www.mdpi.com/article/10.3390/molecules28165985/s1. Table S1: Optimized geometries (coordinates in Å) of studied complexes.
