# Supplementary material for: Main-Group Metal Complexes of Benzene: Predicted Features of Stabilization and Isomerization
Source: Molecules. 2023 Aug 10;28(16):5985. doi: 10.3390/molecules28165985 (PMC10458619; doi:10.3390/molecules28165985)
Supplement: Supplementary file 1 [file molecules-28-05985-s001.zip › molecules-2536054-supplementary.pdf]

Main-group metal complexes of benzene: predicted features of stabilization and isomerization

F. Y. Naumkin

Table S1. Optimized geometries (coordinates in Å) of studied complexes

Be-Bz (p)

6	1.205369130	0.640029930	0.358133970
6	1.200507630	-0.730749230	0.133612110
6	-0.004835440	-1.412070530	0.022354210
6	-1.205358380	-0.722508880	0.135024160
6	-1.200499400	0.648269200	0.359513230
6	0.004837840	1.329500780	0.471420840
1	2.141672320	1.171087180	0.432837800
1	2.132999310	-1.264445690	0.034116390
1	-0.008507410	-2.474996260	-0.163228840
1	-2.141649130	-1.249722480	0.036674780
1	-2.133000430	1.185818330	0.435295950
1	0.008457340	2.396073230	0.634805260
4	-0.000045670	0.412886960	-2.520893860

Be-Bz (c)

6	0.739629250	1.253432060	0.165821630
6	-0.606953460	1.326628720	0.136021630
6	-1.297303990	0.104309410	-0.314799240
6	-0.760945880	-1.087209060	0.384779090
6	0.619045770	-1.162103920	0.416222510
6	1.312495570	-0.037870240	-0.256431230
1	1.367596780	2.060048750	0.512862520

1	-1.158466840	2.197080320	0.457810830
1	-2.366469260	0.123744540	-0.443773420
1	-1.399529880	-1.887755440	0.721925730
1	1.150995440	-2.025871790	0.781285780
1	2.382094240	-0.135151950	-0.337905570
4	-0.010798870	-0.688763420	-1.213421190

Mg-Bz (p)

6	-1.181769870	0.784549910	-0.694570980
6	-1.181769870	0.784549910	0.694570980
6	0.019772300	0.725290400	1.389183460
6	1.221335890	0.666095660	0.694580180
6	1.221335890	0.666095660	-0.694580180
6	0.019772300	0.725290400	-1.389183460
1	-2.115373200	0.827024270	-1.234007620
1	-2.115373200	0.827024270	1.234007620
1	0.019447650	0.720944400	2.468287890
1	2.154513930	0.615134990	1.234044110
1	2.154513930	0.615134990	-1.234044110
1	0.019447650	0.720944400	-2.468287890
12	-0.087236890	-2.392470910	0.000000000

Ca-Bz (p)

6	0.548335620	-1.021427210	1.292973900
6	-0.831905550	-1.026522240	1.129763330
6	-1.380513620	-1.071711650	-0.146199960
6	-0.548722760	-1.109329750	-1.258723680
6	0.831499490	-1.104241030	-1.095572590
6	1.380089750	-1.061567060	0.180462730

1	0.973923630	-0.984931500	2.283528150
1	-1.477031360	-0.993898670	1.993636010
1	-2.451781710	-1.075855080	-0.273545100
1	-0.974315340	-1.141725550	-2.249428600
1	1.476597370	-1.132569490	-1.959603700
1	2.451380820	-1.057749720	0.307775170
20	0.000841920	2.148841160	-0.071807330

Sr-Bz (p)

6	1.218267470	-1.527898040	0.694029110
6	1.218315840	-1.530013490	-0.695987580
6	0.015222260	-1.570535070	-1.390995200
6	-1.187833480	-1.609976800	-0.695946890
6	-1.187914560	-1.607997340	0.693970920
6	0.015091500	-1.566912510	1.388947200
1	2.151586320	-1.490853090	1.233346400
1	2.151733100	-1.495465540	-1.235271960
1	0.015083640	-1.568689520	-2.469541610
1	-2.121483250	-1.639428680	-1.235127880
1	-2.121579440	-1.635313950	1.233257980
1	0.014739640	-1.560379560	2.467505470
38	-0.016041830	1.578469220	0.011814060

Ba-Bz (p)

6	1.243718890	1.744369470	0.695228080
6	1.243700460	1.744380160	-0.695214070
6	0.039566840	1.770884220	-1.390402780
6	-1.164574670	1.797234370	-0.695213090
6	-1.164593400	1.797222840	0.695226980

6	0.039567280	1.770895320	1.390383080
1	2.177163540	1.713727800	1.234444720
1	2.177147800	1.713726720	-1.234442940
1	0.039354110	1.761382390	-2.468783860
1	-2.098466460	1.807254220	-1.234445390
1	-2.098481250	1.807254620	1.234447450
1	0.039353290	1.761383480	2.468773240
56	-0.027595940	-1.243134850	-0.000001420

Ba-Bz (c)

6	-0.677627780	1.525069090	-1.209722860
6	0.693061980	1.518960070	-1.204334990
6	1.437722500	1.235352990	0.007318590
6	0.683496460	1.508103790	1.215543780
6	-0.687194470	1.514237040	1.210143230
6	-1.434319820	1.248237990	-0.003985470
1	-1.201367730	1.739716120	-2.131091040
1	1.225901080	1.729040390	-2.121525630
1	2.498217520	1.425940310	0.012389800
1	1.209084540	1.709979840	2.138750450
1	-1.218194150	1.720736560	2.129211350
1	-2.493052200	1.448419040	-0.007313500
56	-0.008282930	-0.978019300	-0.008465320

Be-Bz-Be (p)

6	0.914354060	-0.031250390	1.043344300
6	-0.448825450	-0.030412070	1.312975340
6	-1.363822490	0.000622190	0.267589980
6	-0.915673050	0.031723390	-1.047383450

6	0.447494170	0.030899800	-1.316994500
6	1.362508500	-0.001156790	-0.271662470
1	1.624968300	-0.055180480	1.855089010
1	-0.796856710	-0.053895400	2.334137560
1	-2.422415480	0.000518450	0.476886620
1	-1.626283390	0.055953390	-1.859106840
1	0.795524940	0.054707680	-2.338147750
1	2.421079150	-0.002862190	-0.481056740
4	0.019333650	2.873265090	0.083524590
4	-0.015158670	-2.873948820	-0.070722980

Be-Bz-Be (s)

6	1.284300900	0.683435170	0.018962040
6	0.056839350	1.516823600	0.163912390
6	-1.076010080	0.584032630	0.583548740
6	-1.078486440	-0.581770720	-0.581175330
6	0.053320440	-1.516942100	-0.164051560
6	1.282746570	-0.686165490	-0.020770960
1	2.239320800	1.189440820	0.000117010
1	0.161358030	2.468977220	0.652874690
1	-2.041812910	1.060625580	0.692097230
1	-2.045469940	-1.056461760	-0.687535380
1	0.155009120	-2.469176880	-0.653449960
1	2.236649610	-1.194289030	-0.002803890
4	-0.476494290	-0.851695490	1.241223180
4	-0.477908380	0.852832570	-1.240135640

Mg-Bz-Mg (p)

6	-0.294294470	0.000098970	-1.358489090

6	-1.328457900	0.008185390	-0.430157490
6	-1.041669640	0.008482360	0.929588470
6	0.279355000	0.000694200	1.360891830
6	1.313581270	-0.007375490	0.432620240
6	1.026722230	-0.007676400	-0.927125890
1	-0.517056980	-0.000110270	-2.414315040
1	-2.354177910	0.014731670	-0.765248600
1	-1.844696520	0.015268980	1.650449500
1	0.502007920	0.000991220	2.416756010
1	2.339288610	-0.013794190	0.767721150
1	1.829761660	-0.014340830	-1.648008590
12	0.018875070	3.178985140	-0.003893470
12	-0.001182400	-3.180143040	0.000957600

Ca-Bz-Ca (p)

6	1.203842460	0.006170320	0.695077070
6	1.203842460	0.006170320	-0.695077070
6	-0.000071120	0.001113210	-1.390219990
6	-1.203975970	-0.004025260	-0.695104560
6	-1.203975970	-0.004025260	0.695104560
6	-0.000071120	0.001113210	1.390219990
1	2.138323580	0.010587350	1.234572390
1	2.138323580	0.010587350	-1.234572390
1	0.000176970	0.001045020	-2.469247930
1	-2.138530000	-0.008824340	-1.234442060
1	-2.138530000	-0.008824340	1.234442060
1	0.000176970	0.001045020	2.469247930
20	-0.011189460	-3.391484100	0.000000000
20	0.024494120	3.409516460	0.000000000

Ca-Bz-Ca (s)

6	-0.007161610	-1.416719440	-0.004210800
6	1.227507040	-0.758205640	-0.003549150
6	1.235063250	0.745769470	-0.003815050
6	0.007161610	1.416719440	-0.004210800
6	-1.227507040	0.758205640	-0.003549150
6	-1.235063250	-0.745769470	-0.003815050
1	-0.012656670	-2.498457120	-0.004609920
1	2.147003890	-1.315341930	-0.003995760
1	2.160138070	1.293577030	-0.003260360
1	0.012656670	2.498457120	-0.004609920
1	-2.147003890	1.315341930	-0.003995760
1	-2.160138070	-1.293577030	-0.003260360
20	0.000000000	0.000000000	2.186618270
20	0.000000000	0.000000000	-2.195067230

Sr-Bz-Sr (p)

6	1.203839830	-0.025375240	0.692536570
6	1.204698810	-0.022318300	-0.697973620
6	0.000543450	-0.002619040	-1.392878350
6	-1.203670390	0.014936040	-0.697589310
6	-1.204591330	0.012575670	0.693141360
6	-0.000238220	-0.007684500	1.388024560
1	2.137750780	-0.041771400	1.232641590
1	2.138787010	-0.035638440	-1.237240000
1	-0.000784730	-0.000296410	-2.471850860
1	-2.137163520	0.030328420	-1.238149280
1	-2.138944320	0.026993080	1.232479740

1	0.001437090	-0.009653190	2.466897070
38	-0.061422810	-3.384236630	0.021074110
38	0.065558120	3.395838180	0.008886410

Sr-Bz-Sr (s)

6	-0.693416540	-0.003551900	-1.262603560
6	0.704507490	-0.001516020	-1.224842480
6	1.440353420	0.001623220	-0.035679470
6	0.693538590	0.002333380	1.262945280
6	-0.704323690	0.000243540	1.225106120
6	-1.440208680	-0.002860930	0.035966110
1	-1.215968760	-0.005679330	-2.203081970
1	1.244068890	-0.002048370	-2.162942110
1	2.516045630	0.003043820	-0.057139090
1	1.216176380	0.004251990	2.203280700
1	-1.243781200	0.000736930	2.163347640
1	-2.515865670	-0.004075660	0.057294810
38	0.003356440	-2.364447230	0.003526360
38	-0.005138980	2.363105420	-0.004896130

Ba-Bz-Ba (p)

6	1.211549000	0.048838840	0.696121600
6	1.211542800	0.048841950	-0.696115800
6	0.005857140	0.057625010	-1.392230780
6	-1.199801150	0.069436930	-0.696121320
6	-1.199795160	0.069437350	0.696123710
6	0.005859950	0.057622570	1.392235300
1	2.145627500	0.041384970	1.235393490
1	2.145612910	0.041391920	-1.235402320

1	0.005822260	0.056622090	-2.470831090
1	-2.133850790	0.077919850	-1.235435660
1	-2.133838470	0.077919070	1.235448550
1	0.005786780	0.056618530	2.470835230
56	-0.022914990	-3.122482020	-0.000006160
56	0.030994590	3.239068630	-0.000014740

Ba-Bz-Ba (s)

6	-1.414118300	0.001484160	-0.004105600
6	-0.750141060	0.000427810	1.224997430
6	0.737336370	-0.003081620	1.228232840
6	1.406197510	-0.003821010	0.001756820
6	0.742430140	-0.001662090	-1.227432920
6	-0.745016800	0.001772580	-1.230403130
1	-2.496259970	0.002988400	-0.006357760
1	-1.306155170	0.000577780	2.146682100
1	1.289386560	-0.005262200	2.152182190
1	2.488433190	-0.006462210	0.003948590
1	1.298295850	-0.001900810	-2.149097410
1	-1.297201120	0.001844610	-2.154386040
56	0.000219660	2.526152960	0.000499960
56	-0.009255990	-2.527284580	-0.001774820
